# Gallic acid released by a layered double hydroxide-coated scaffold of hydroxyapatite and β-tricalcium phosphate inhibits the osteoclast formation *In Vitro*

**DOI:** 10.1016/j.bbiosy.2025.100119

**Published:** 2025-08-20

**Authors:** Chiara Suvieri, Maria Bastianini, Stefano Pagano, Lorella Marinucci, Valeria Ambrogi, Leonardo Leonardi, Carmela Conte, Maria Teresa Pallotta, Bernard Fioretti, Giovanna Traina, Michele Sisani, Maria Laura Belladonna

**Affiliations:** a Department of Medicine and Surgery, University of Perugia, 06129 Perugia, Italy; b R&D Department, Prolabin&Tefarm S.r.l., 06134 Ponte Felcino, Perugia, 06134 Italy; c Department of Pharmaceutical Sciences, University of Perugia, 06123 Perugia, Italy; d Department of Veterinary Medicine, University of Perugia, 06121 Perugia, Italy; e Department of Chemistry, Biology and Biotechnologies, University of Perugia, 06123 Perugia, Italy

**Keywords:** Osteoclasts, Alveolar bone loss, Gallic acid, LDH, Bone substitute, Drug delivery system

## Abstract

Following dental extraction, alveolar bone loss, driven by the osteoclast (OC) bone-eroding cells, is a relevant concern in dental practice since it could compromise the possibility of installing dental implants. This study aimed to develop a drug delivery system releasing the antiosteoclastogenic molecule gallic acid (GA) at the alveolar bone level to control the dysregulated balance between OCs and bone-building osteoblasts and thus delay bone erosion. We functionalized small blocks of the hydroxyapatite- and β-tricalcium phosphate-based RIGENERA BTK BCP biomaterial with layered double hydroxide (LDH) and GA (RIG_LDH-GA). By the in vitro model of Receptor Activator of Nuclear factor Kappa-Β Ligand (RANKL)-induced osteoclastogenesis in RAW 264.7 macrophages, we demonstrated that the conditioned medium (CM) obtained after 1-day incubation with RIG_LDH-GA contrasts the OC formation in a dose-dependent manner until a complete inhibition at the highest tested dose, while the unfunctionalized control (RIG) is ineffective. TRAP enzyme activity, OC marker gene expression, and bone resorption activity confirmed the antiosteoclastogenic effect of RIG_LDH-GA CM. Moreover, the expression of RANK (the RANKL’s receptor), otherwise induced by RANKL treatment, was reduced to the untreated control extent, consistent with the decreased expression of the transcription factors c-Fos and NFATc1, activated downstream in the RANK signaling pathway and inducing RANK itself. Thus, since GA released by the RIG_LDH-GA system effectively exerted an antiosteoclastogenic effect, RIGENERA BTK BCP functionalization with LDH and GA likely appears to be an osteoprotective upgrade of this biomaterial, already possessing bone regenerative properties, and might find successful clinical application in preventing osteoclast-mediated alveolar bone loss.

## Highlights

• Alveolar bone loss in the socket of an extracted tooth is caused by osteoclast hyperactivity.
• A synthetic bone substitute coated with a lamellar solid intercalating gallic acid was prepared.
• Gallic acid was efficiently released in the conditioned medium by this drug delivery system.
• The conditioned medium inhibited osteoclast formation and functions in an in vitro cell model.
• This strategy might find successful application in postextractive alveolar ridge preservation.

## Graphical abstract

**Figure:**
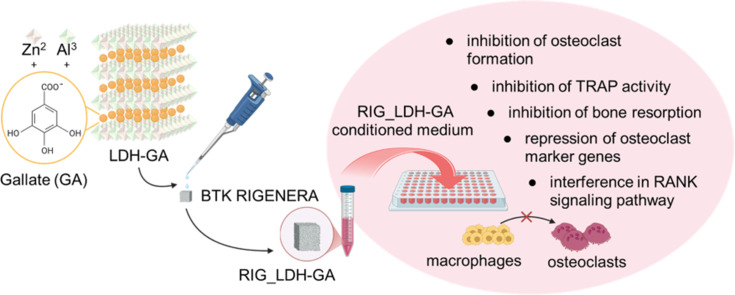

## 1 Introduction

Periodontal disease and dental caries are among the primary causes of tooth extraction, a common dental procedure. Following extraction, the alveolar bone and surrounding soft tissues undergo a dynamic healing process known as socket healing, which begins immediately after tooth removal and typically continues for up to six months [1]. In the absence of intraosseous stimulation, normally provided by periodontal ligament fibers [2], the physiological balance between bone-forming and bone-resorbing cells becomes disrupted. As a result, bone resorption predominates, leading to rapid and substantial loss of alveolar bone volume [3].

Bone remodeling is a tightly regulated process involving three sequential phases: bone resorption by osteoclasts (OCs) that degrade the bone surface, a reversal phase, and bone formation by osteoblasts (OBs). OCs originate from the macrophage lineage of hematopoietic stem cells in response to Receptor Activator of Nuclear factor Kappa-Β Ligand (RANKL) stimulation [4], while OBs derive from mesenchymal stem cells through differentiation into osteoprogenitors and preosteoblasts [5]. When osteoclastic activity is excessive and not adequately compensated by osteoblastic bone formation, the imbalance promotes bone loss and contributes to the development of osteoporosis [6].

In the oral cavity, progressive alveolar bone resorption after tooth extraction or loss poses a major challenge for implant-based rehabilitation due to reduced bone height and volume [7]. A common clinical scenario is the crestal migration of the maxillary sinus floor, often necessitating sinus floor elevation procedures to restore adequate bone height [[8], [9], [10]]. For sinus lift involves lifting the sinus membrane and filling the created space with bone graft materials—autologous, xenogeneic, or synthetic—including calcium carbonate, calcium phosphate, hydroxyapatite (HA), and β-tricalcium phosphate (β-TCP) [11]. These biomaterials significantly influence resorption kinetics and implant success.

Among inorganic biomaterials, HA and β-TCP are widely used due to their osteoconductive properties and chemical similarity to natural bone [12,13]. HA provides mechanical strength and osteoinductive potential [14], while β-TCP, which undergoes faster biodegradation and resorption due to its lower Ca/P ratio [15], offers a resorbable, porous structure conducive to cell adhesion and vascularization [16]. Biphasic calcium phosphate (BCP), which combines HA and β-TCP, achieves a balance between structural stability and timely resorption [17].

RIGENERA BTK BCP is a commercially available BCP scaffold where HA and β-TCP are uniformly distributed. As bone forms, the HA component provides mechanical strength under compressive forces supporting early bone matrix deposition and maintaining scaffold integrity [18,19], whereas β-TCP contributes to the formation of an interconnected porous structure, facilitating vascular ingrowth and tissue integration [15,20,21]. This combination supports the rapid development of a vital, newly formed bone matrix by maintaining a balance between material resorption and scaffold stability [17].

Layered double hydroxides (LDH), also referred to as hydrotalcites, are inorganic lamellar materials composed of positively charged layers of mixed bivalent and trivalent metal hydroxides, intercalated with charge-balancing anions [22]. These structures exhibit excellent biocompatibility and unique physicochemical properties, making them increasingly attractive for applications in tissue engineering [23], particularly in the field of bone regeneration [[24], [25], [26], [27]]. The lamellar architecture of LDH can be precisely engineered by varying the metal ion composition and intercalated molecules, enabling their use as drug delivery systems [28,29]. Different types of LDH are increasingly explored for bone regeneration due to their ion-exchange capability, functionalization with various bioactive agents and ability to sustain bioactive release. For instance, zinc ions (Zn²⁺) have been incorporated due to their osteogenic and antibacterial effects [30]; alendronate has been successfully intercalated and released as a bone-protective agent [19]; MgAl-LDH nanoparticles were able to enhance osteogenic differentiation via LGR5/β-catenin activation and reduce inflammation through NF-κB inhibition [31]; MgAlEu-LDH/HA scaffolds promoted bone mineralization in vivo through synergistic Mg²⁺ and Eu³⁺ release [13]; and LDH coatings on bioactive glass incorporating selenium improved both osteogenesis and antibacterial activity [29]. Such systems combine structural support with biochemical cues, making them promising candidates for next-generation bone grafts.

Gallic acid (GA), or 3,4,5-trihydroxybenzoic acid, is a small phenolic compound widely recognized for its anti-inflammatory [32] and antioxidant properties across various models of oxidative stress, which are implicated in neurological and cardiovascular diseases [33]. In addition to these effects, GA has demonstrated protective activity in bone metabolism. Specifically, in models of RANKL-induced osteoclastogenesis using bone marrow-derived macrophages and RAW 264.7 cells, GA has been shown to inhibit osteoclast differentiation and function by blocking the Akt, ERK, and JNK signaling pathways and by downregulating the expression of osteoclastogenesis-associated markers [34,35]. Furthermore, its ability to prevent bone loss in vivo has been validated in an ovariectomized mouse model, highlighting its potential as an osteoprotective agent [34].

The aim of the present study is to improve the performance of the commercial bone graft material RIGENERA BTK BCP by functionalizing its surface with a ZnAl-LDH loaded with GA (LDH-GA), thereby creating a novel antiosteoclastogenic delivery system for potential application in socket preservation following tooth extraction. The LDH-GA system was synthesized, thoroughly characterized, and subsequently deposited onto small blocks of RIGENERA BTK BCP. To assess its release profile, a conditioned medium (CM) was prepared by incubating the resulting composite (referred to as RIG_LDH-GA**)** in cell culture medium, and the concentration of GA released was quantified. Finally, the inhibitory effect of RIG_LDH-GA CM on osteoclast formation was evaluated using an in vitro model of RANKL-induced osteoclastogenesis in RAW 264.7 cells. To the best of our knowledge, this is the first study to investigate a ZnAl-LDH system loaded with GA in combination with HA and β-TCP for its antiosteoclastogenic potential.

## 2 Materials and methods

### 2.1 Reagents and antibodies

Zinc nitrate hexahydrate [Zn(NO_3_)_2_ × 6H_2_O] (98 %), aluminum nitrate nonahydrate [Al(NO_3_)_3_ × 9H_2_O] (98 %), urea (99 %), NaOH (98 %), sodium acetate, GA (97.5–102.5 %), acetonitrile, trifluoroacetic acid, HNO_3_, and HCl were purchased from Sigma-Aldrich (St. Louis, MO, USA).

Recombinant murine RANKL was from PeproTech (London, UK). For cell viability tests, 3-(4,5-dimethylthiazol-2-yl)-2,5-diphenyltetrazolium bromide (MTT) was purchased from Sigma-Aldrich (St. Louis, MO, USA). As a tartrate-resistant acid phosphatase (TRAP) staining reagent, Acid Phosphatase Kit 387-A was used (Sigma-Aldrich, St. Louis, MO, USA). TRAP reaction buffer was prepared as described by the manufacturer’s instructions (Sigma-Aldrich, St. Louis, MO, USA). The following primary antibodies were used for immunoblotting: mouse NFATc1 (7A6) monoclonal antibody (#MA3–024, Invitrogen, Carlsbad, CA, USA), rabbit c-Fos (9F6) monoclonal antibody (#2250, Cell Signaling Technology, Danvers, MA, United States), rabbit RANK antibody (#4845, Cell Signaling Technology, Danvers, MA, United States), and mouse β-tubulin (AA2) monoclonal antibody (#T8328, Sigma-Aldrich, St. Louis, MO, USA), rabbit Acp5 polyclonal antibody targeting TRAP protein (#PA5–106914, Invitrogen, Carlsbad, CA, USA), rabbit Ctsk polyclonal antibody (#PA5–102483, Invitrogen, Carlsbad, CA, USA), mouse Mmp9 (5G3) monoclonal antibody (#MA5–15886, Invitrogen, Carlsbad, CA, USA) rabbit Calcr polyclonal antibody (#20868–1-AP, Proteintech, Rosemont, IL, USA).

### 2.2 Preparation of LDH-GA

An LDH containing ZnAl was synthesized directly in the nitrate form (LDH—NO_3_) using the urea method [36]. The LDH—NO_3_ was used to pre-intercalate acetate anions. One gram of LDH—NO_3_ was dispersed in 2.7 mL of a water solution of sodium acetate (1 M, 0.8 g/mL). The dispersion was stirred for 24 h at room temperature in a closed vessel under nitrogen. The lamellar solid intercalating acetate (LDH-ACE), recovered by centrifugation, was washed 3 times with water, dried in the oven at 60 °C, and used for the preparation of the GA-containing solid. Sixty mL of a sodium gallate water solution (0.13 M) has been prepared by adding an equimolar amount of 1 M NaOH. One gram of LDH-ACE was dispersed in this solution under nitrogen in a closed vessel under magnetic stirring for 3 h. The obtained LDH-GA solid was recovered by centrifugation, washed 2 times with water, and freeze-dried.

### 2.3 Analytical and structural characterization techniques

Twenty mg of LDH—NO_3_ were dissolved using few drops of concentrated HNO₃. The solution was brought to a volume of 250 mL with deionized water. Zn and Al content in this solution were determined by Inductively Coupled Plasma-Optical Emission Spectrometer (ICP-OES) (Perkin Elmer, Avio 200 Waltham, MA, USA).

X-Ray Powder Diffraction (XRPD) patterns were recorded with a Bruker D2 Phaser diffractometer operating at 30 kV and 15 mA, a step size of 0.02 (2θ degrees), and a time per step of 1 s, using the Cu Kα radiation (1.54 Å) and multistrip LYNXEYE SSD160 detector.

The gallate content in LDH-GA was determined on the solution obtained after having dissolved 60 mg of the solid in a mix of 3 mL of 2 N HCl and 40 mL of acetonitrile:water (1:1), then diluted with water to 100 mL. The following HPLC method was used: Zorbax column SB C18, 4.6 × 250 mm, 5-micron (Agilent P.N. 880,975–902); mobile phase: (acetonitrile + 0.1 % v/v trifluoroacetic acid) / (water + 0.1 % v/v trifluoroacetic acid) = 20/80, isocratic; injected volume: 20 µL; column temperature: 35 °C; flux: 1 ml/min; detector: VWD lambda 272 nm; total analysis time: 6–10 min; retention time of GA: 3.05 min. The method was also used to determine the kinetics of GA release.

Powders of GA, LDH-ACE, and LDH-GA were dispersed in KBr pellets and Fourier Transform Infrared Spectroscopy (FT-IR) spectra of them were recorded at room temperature using a FT-IR-4600 Jasco (JASCO UK Limited, Heckmondwike, UK). Typically, each spectrum was obtained in the spectral region from 400 to 4000 cm⁻¹.

The LDH-GA sample morphology was investigated with a Phenom PRO G6 Desktop Scanning Electron Microscope (SEM) (Bruker, Billerica, MA, USA). SEM micrographs were collected by depositing the sample on a stub holder and after a sputter coating with gold for 60 s.

### 2.4 RIGENERA BTK BCP functionalization

RIGENERA BTK BCP was kindly provided by Biotec S.r.l. (Vicenza, Italy) in the form of small blocks measuring approximately 1 × 1 × 0.5 cm and weighing about 250 mg. Each RIGENERA BTK BCP block was weighted and an amount of LDH-GA corresponding to 10 % of each block weight was prepared. The LDH-GA was finely ground and dispersed in a small amount of water (0.5 mg/µL). The functionalization was carried out by depositing the water suspension on the surface of a RIGENERA BTK BCP block. The deposition of the LDH-GA on the solid block was favored under vacuum in a small Buckner. Then, blocks were left drying at room temperature.

### 2.5 Kinetics of GA release

The kinetics study of GA release was conducted in duplicate by incubating a 250-mg functionalized block in 2 mL of physiological solution (NaCl 0.9 %) in a closed vessel at 37 °C for 48 h without stirring. At the indicated time points, the incubation solution was removed and replaced with the same volume of pre-warmed physiological solution. GA content was determined by HPLC, as described above.

### 2.6 Cell cultures and treatments

The murine macrophage cell line RAW 264.7 was obtained from the American Type Culture Collection (ATCC, Manassas, VA, USA). Cells were cultured according to standard procedures in RPMI-1640 medium (Gibco Thermo Fisher Scientific, Waltham, MA, USA), supplemented with 10 % heat-inactivated fetal bovine serum (FBS), 2 mM of l-glutamine, and antibiotics (100 U/mL penicillin and 100 μg/mL streptomycin), and used the day after vial thawing. Incubations were performed at 37 °C in a 5 % CO₂ atmosphere and humidified air. For the evaluation of bone resorption activity, RAW 264.7 cells were cultured in phenol red-free MEMα (Gibco Thermo Fisher Scientific, Waltham, MA, USA) supplemented with 10 % FBS, 2 mM l-glutamine, and antibiotics (100 U/mL penicillin and 100 μg/mL streptomycin). Differentiation in OCs was achieved by culturing RAW 264.7 cells with RANKL (100 ng/mL) for 5 days, replacing culture medium containing stimuli on the third day. After UV sterilization, RIG_LDH-GA and RIG CM were prepared by incubating 250-mg RIGENERA BTK BCP blocks functionalized with LDH-GA or unfunctionalized, respectively, in 2 ml of cell culture medium for 24 h at 37 °C. Briefly, 1 × 10^4^ cells/cm^2^ were seeded the day before treatment with RANKL in the absence or presence of scalar amounts of RIG_LDH-GA or RIG CM, the latter used as a negative control. Cells were cultured in 96-well plates (3 × 10^3^ cells in 100 μL/well) to evaluate cell viability, OC number, and TRAP activity, or in 48-well plates for qPCR and Western blotting analysis (7.2 × 10^3^ cells in 240 μL/well, or 5 × 10^4^ cells in 500 μL/well for c-Fos and NFATc1, or RANK immunoblot, respectively).

### 2.7 Cell viability assay

After 5 days of culture with either the RIG_LDH-GA or RIG CM in the presence of RANKL, RAW 264.7 cell viability was measured by MTT assay. Culture medium was removed, and adherent cells were incubated for 4 h at 37 °C with 110 μL of medium containing 50 μg of MTT. After the addition of 100 μL of solubilization buffer (SDS 10 % in HCl 0.01 M) to each well and an overnight incubation at 37 °C, absorbance was read at 570 nm by a UV/visible spectrophotometer (TECAN, Thermo Fisher Scientific, Waltham, MA, USA). The assay was performed in triplicate for each concentration.

### 2.8 F-actin ring-formation assay

After OCs differentiation, cells were fixed with 4 % formaldehyde (10 % formalin, neutral buffered) (Merk, Darmstadt, Germany) in PBS 1x for 20 min and then stained with 4 µg/ml TRITC-labelled phalloidin (Merk, Darmstadt, Germany) for 30 min at room temperature. Nuclei were stained with Hoechst 33,342 (Thermo Scientific, Waltham, MA, USA) for 10 min at room temperature. Fluorescence microscopy images were obtained by a Nikon ECLIPSE Ti inverted microscope equipped with an X-Light V2 LFOV spinning disk, and a large image of the whole well was captured and used for the total count of OCs for each condition.

### 2.9 TRAP staining and activity

TRAP-positive cells and soluble TRAP activity were assessed in RANKL-induced differentiation cultures by Acid Phosphatase Kit 387-A (Sigma-Aldrich, St. Louis, MO, USA) according to the manufacturer’s protocol. Briefly, after incubation of RAW 264.7 cells with either RIG_LDH-GA or RIG CM in the presence of RANKL, TRAP activity was revealed at both the supernatant and cell levels. Thus, 80 μL of harvested supernatants were added to 120 μL of 1.7x TRAP reaction buffer, and, after 3 h of incubation at 37 °C, absorbance at 540 nm was measured using a UV/visible spectrophotometer (TECAN, Thermo Fisher Scientific, Waltham, MA, USA). Adherent cells were fixed and stained by TRAP reagent in light-protected conditions at 37 °C for 1 h. Images of TRAP-positive cells were captured under a bright-field light microscope (EVOS M5000, Thermo Fisher Scientific, Waltham, MA, USA).

### 2.10 Resorption pit formation assay

The assay was performed in 96-well plates pre-coated with carbonate apatite (CaP) in accordance with the Bone Resorption Assay Kit protocol (CSR-BRA-S96KIT, Cosmo Bio Co., LTD). Briefly, RAW 264.7 cells were seeded (2 × 10^3^/200 µl/well) and allowed to adhere overnight. The supernatant was replaced with culture medium containing RANKL (100 ng/ml), alone or in combination with 100 μL of RIG_LDH-GA or RIG CM. After 6 days of incubation at 37 °C, cells were removed by treating wells with 5 % sodium hypochlorite (100 μL/well). After washing the plates, the pits were photographed under a bright-field light microscope (EVOS M5000, Thermo Fisher Scientific, Waltham, MA, USA), and their area was measured by the thresholding tool of ImageJ software (v1.54p).

### 2.11 Real-time PCR

After total RNA extraction by TRIzol (Invitrogen, Carlsbad, CA, USA) and reverse transcription to cDNA by QuantiTect Reverse Transcription Kit (Qiagen, Hilden, Germany), real-time PCR was performed using SYBR Green (Bio-Rad, Hercules, CA, USA) detection and specific primers (23). Values were calculated as the ratio of the specific gene to *Gapdh* expression, as determined by the relative quantification method (ΔCT; means ± SD of triplicate determination) [37]. Data are analyzed by the comparative 2^−ΔΔCt^ method to determine the gene expression in treated samples as fold change relative to the RANKL calibrator, taken as unitary reference (fold change = 1).

### 2.12 Western blotting

Cells were lysed with Laemmli buffer containing 2-mercaptoethanol, and proteins were separated on 10 % acrylamide gels by SDS/PAGE. After protein transfer onto a nitrocellulose membrane by the Trans-Blot Turbo System (Bio-Rad, Hercules, CA, USA) at 25 V for 10 min, membranes were blocked with 3 % BSA in TBST for 1 h at room temperature. Then, membranes were incubated with primary antibodies at 4 °C overnight, followed by incubation for 1 h with an appropriate horseradish peroxidase-conjugated antibody (Merk Millipore, Burlington, MA, USA). The signals were visualized via enhanced chemiluminescence (ECL, Bio-Rad, Hercules, CA, USA) detection, acquired by the ChemiDoc MP Imaging System (Bio-Rad, Hercules, CA, USA). β-tubulin was used as a loading control.

### 2.13 Statistical analysis and IC_50_ calculation

The in vitro determinations are mean ± SD from at least three independent experiments. Counts were performed by three independent operators blind to the treatments. Representative experiments and images are shown unless stated otherwise. Statistical significance was determined by the ANOVA one-way or two-way analysis. The IC_50_ values were calculated by the non-linear regression method [log(inhibitor) vs. normalized response—variable slope] using GraphPad Prism Version 10.4.2 software. p-values < 0.05 were considered statistically significant.

## 3 Results

### 3.1 Bone substitute functionalization and characterization

**Fig. 1:**
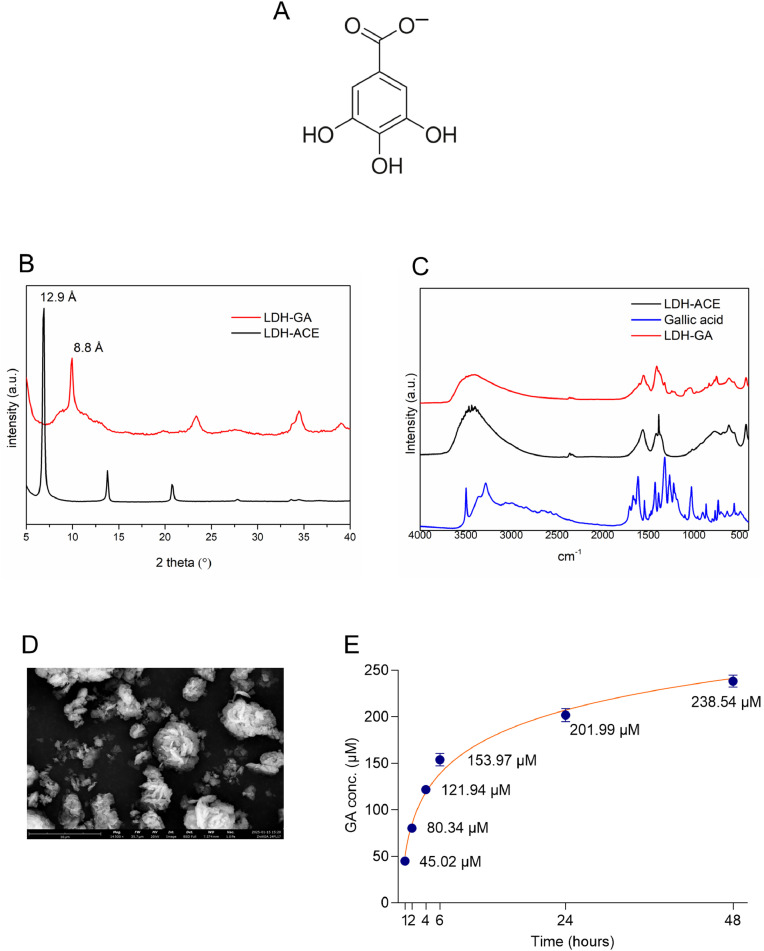
Characterization of LDH intercalating GA. (**A**) Structural formula of monoanionic GA. (**B**) XRPD of LDH-ACE (black) and LDH-GA (red). (**C**) FT-IR analysis of LDH-ACE (black), GA (blue) and LDH-GA (red). (**D**) SEM micrograph of LDH-GA, acquired with a BSD full detector; magnification 14,500x. (**E**) Kinetics of GA released in 2 mL of physiological solution after the indicated times at 37 °C by 250-mg blocks of RIGENERA BTK BCP functionalized with LDH-GA (RIG_LDH-GA). Data are mean values ± SD of two independent determinations, each conducted in duplicate.

Functionalization of the RIGENERA BTK BCP biomaterial with GA-intercalated LDH was achieved by exchanging the anions in the interlayer region of the LDH lamellar structure through intercalation reactions [38]. The pristine ZnAl-based material was intercalated with acetate anions, which were subsequently exchanged with monodeprotonated GA anionic molecules (i.e., gallate molecules) (Fig. 1**A**). The acetate form was chosen as the pristine material to facilitate the ion exchange process, following several unsuccessful intercalation attempts using the nitrate form. In our settings, the amount of NaOH added to the GA solution led to the deprotonation of only the carboxyl group, producing a monoanionic molecule suitable for exchanging the acetate previously intercalated in the LDH. Thus, a suspension of LDH-ACE was contacted with an aqueous solution of monoanionic GA, magnetically stirred for 3 h, and washed twice with water. The resulting LDH-GA solid was freeze-dried and characterized by XRPD analysis to confirm the intercalation. As shown in Fig. 1**B**, the d-spacing in the starting material was 12.9 Å, a distance previously attributed to the presence of acetate in the interlayer region [39]. After contact with GA for anion exchange, the interlayer distance in the LDH-GA sample decreased to 8.8 Å, consistent with a flat monolayer of gallate [40]. The following formula was assigned to the LDH-GA sample based on ICP-OES and HPLC analysis:

$$\left[Zn_{0.72}Al_{0.28}{\left(\text{OH}\right)}_{2}\right]{\left(\text{GA}\right)}_{0.28}\times H_{2}O$$

The GA-loaded amount corresponded to 30.4 % of LDH mass, confirming complete anion exchange, with monoanionic GA replacing acetate. The successful intercalation of GA into the ZnAl-LDH interlayer was also analyzed by FT-IR spectroscopy for GA, LDH, and LDH-GA (Fig. 1**C**). By comparing the FT-IR spectrum of LDH-GA with those of GA and LDH-ACE, the intercalation of gallate into the lamellar structure can be confirmed. Specifically, the FT-IR spectrum of GA shows two bands at 3500 cm⁻¹ and 3300 cm⁻¹, corresponding to the stretching vibrations of the phenolic hydroxyl and carboxylic groups, respectively [41]. These bands are absent in the LDH-GA spectrum: the band at 3300 cm⁻¹ disappears due to the deprotonation of GA and subsequent salt formation, while the band at 3500 cm⁻¹ is no longer visible as a result of hydrogen bonding involving the phenolic –OH group within the interlayer region of the LDH. The band at approximately 1386 cm⁻¹, attributed to –CH– bending vibrations of the benzene ring, is evident in both the GA and LDH-GA spectra. In the 800–400 cm⁻¹ region, the LDH-ACE and LDH-GA spectra exhibit translational modes associated with Al–OH and Zn–OH groups, located at approximately 427, 551, 612, and 697 cm⁻¹ [42].

The LDH-GA solid was observed by SEM, and the corresponding micrograph (Fig. 1**D**) shows the typical lamellar structure with a classical desert rose-like morphology [36].

RIGENERA BTK BCP blocks were weighed before and after functionalization and characterized by XRPD analysis to confirm the presence of the layered solid (Fig. S1). The spectrum of RIG confirms the presence of HA and β-TCP. Since, during the functionalization, LDH was deposited on the surface, the HA and β-TCP signals are completely covered by those of LDH-GA.

Thus, XRPD, ICP, HPLC and FT-IR analyses validated the presence of gallate in the freeze-dried LDH-GA and confirmed the solid as suitable to be afterwards used in the preparation of RIGENERA BTK BCP blocks coated with LDH-GA (RIG_LDH-GA).

RIGENERA BTK BCP is a synthetic, porous biomaterial composed of 30 % low-reabsorption HA and 70 % rapidly reabsorbed β-TCP. This bone substitute, supplied in the form of small blocks measuring 1 × 1 × 0.5 cm, was functionalized with LDH-GA by depositing a highly concentrated (0.5 mg/μL) aqueous suspension of LDH-GA onto the surface of a RIGENERA BTK BCP block, which was then dried. The resulting RIG_LDH-GA solid was analyzed in a time-course experiment to evaluate its GA-releasing ability and thus assess the therapeutic potential of this system. The amount of GA released into 2 mL of physiological solution at 37 °C after 1, 2, 4, 6, 24, or 48 h of incubation by a 250-mg block of RIGENERA BTK BCP functionalized with LDH-GA was analyzed via HPLC. In Fig. 1**E**, the results for the indicated time points are reported as micromoles per liter (μmol/L) ± SD of GA released from the RIGENERA block functionalized with LDH-GA. Over time, the GA concentration increased quickly before and slowly after 24 h.

These results demonstrate that GA can be efficiently and slowly released by the RIG_LDH-GA system, with approximately 1 % of the total loaded GA released in the first 24 h. Because the release kinetics trend indicates the 24-h time point as borderline between the fast- and slow-release phases, we have chosen this time point for the preparation of the CM afterwards tested in cell cultures of RANKL-induced osteoclastogenesis.

### 3.2 Inhibition of OC formation

The releasing system RIGENERA BTK BCP, functionalized with LDH and GA, was developed with the aim of achieving a local effect to counteract bone loss. Before evaluating the potential inhibitory activity exerted by RIG_LDH-GA CM on the RANKL-induced differentiation process leading to bone-eroding OCs, the absence of any toxic effect was assessed under the same conditions. To this purpose, RIG_LDH-GA CM—obtained by incubating a 250-mg functionalized RIGENERA BTK BCP block in 2 mL of culture medium for 24 h—was tested in 5-day cultures of RAW 264.7 cells treated with RANKL. RIG CM, prepared in the same way, was also included in the experiment as negative control. To better evaluate the potential dose-dependency of the observed effects on cell viability, RIG_LDH-GA and RIG CMs were diluted 1:2 and tested in a range of volumes, corresponding to a gradient of GA content. The MTT assay revealed that the viability of RAW 264.7 cells was unaffected after five days of co-treatment with RANKL and increasing volumes (ranging from 3.1 to 50 µL/well) of either RIG or RIG_LDH-GA CM, the latter giving a GA final concentration of 12.5 to 100 μM (Fig. S2). For both treatments, at all tested dilutions, no significant variation in cell viability was observed compared to the RANKL control (black histogram), with values close to 100 % and consistently above 75 %. These results support the conclusion that both RIG_LDH-GA CM and its negative control, RIG CM, are safe and thus suitable for further evaluation in RANKL-treated RAW 264.7 cell cultures to assess potential inhibitory effects on OC formation.

**Fig. 2:**
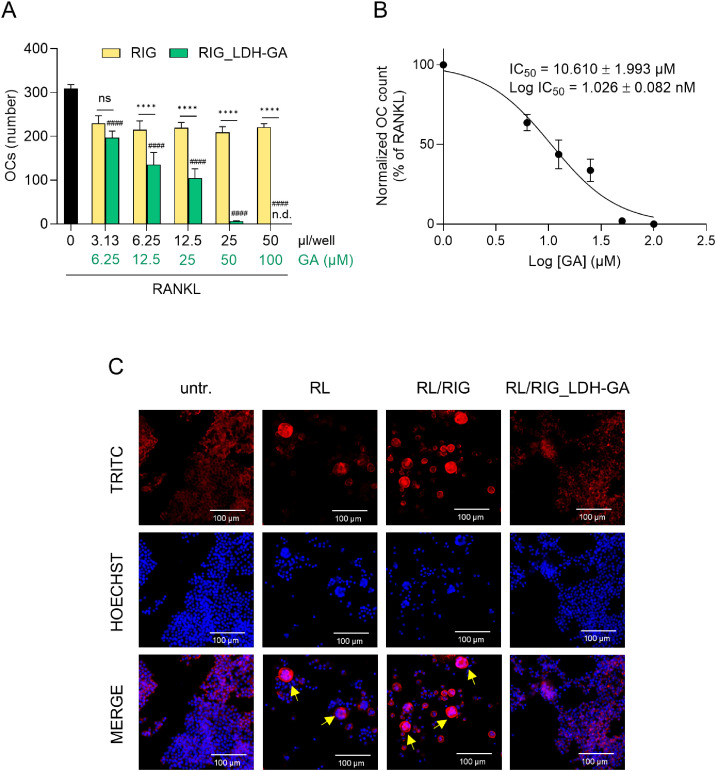
OC count in cultures of RAW 264.7 cells treated with RANKL and RIG_LDH-GA or RIG CM. (**A**) Number of OCs in 5-day cultures of RAW 264.7 cells treated with RANKL alone (100 ng/mL) (black bar) or in combination with either RIG_LDH-GA (green bars) or RIG (yellow bars) CM. For each sample treated with different volumes of RIG_LDH-GA CM, GA content is reported as the final concentration reached in the 96-well plate cell culture (100 μL/well). The number of OCs, counted in the whole area of each well, is reported as a mean value ± SD of three distinct counts. Only actin ring-positive cells having three or more nuclei were considered OCs. ****, *p* < 0.0001, RANKL/RIG_LDH-GA-treated versus RANKL/RIG-treated (two-way ANOVA test); ^####^, *p* < 0.0001, RANKL/RIG_LDH-GA-treated versus RANKL-differentiated control (one-way ANOVA test); n.d., not detectable. (**B**) A concentration-response curve was obtained to determine the IC_50_ of GA released by RIG_LDH-GA CM. The IC_50_ value was calculated using non-linear regression analysis. (**C**) Fluorescent images obtained by staining nuclei in blue with Hoechst and actin rings in red with phalloidin-TRITC. Cells were untreated (untr.), cultured with RANKL alone (RL), or in combination with 50 µL/well of either RIG_LDH-GA (RL/RIG_LDH-GA) or RIG (RL/RIG) CM. Merged images allow identifying OCs defined as cells containing three or more nuclei (blue) and delimited by the actin ring (red). Some OCs are indicated by arrows. Fluorescence microscopy images were obtained by a Nikon ECLIPSE T*i* inverted microscope equipped with an X-Light X-Light V2 LFOV spinning disk; a large image of the whole well was captured and used for the total count of OCs for each condition.

The antiosteoclastogenic effect of RIG_LDH-GA CM was investigated in vitro using the model of RANKL-induced differentiation of RAW 264.7 macrophages into OCs. The number of OCs was evaluated as a marker of the degree of differentiation in cultures incubated for 5 days with both RANKL and a range of doses (3.13 to 50 μL/well) of RIG_LDH-GA CM (GA final concentration of 6.25 to 100 μM). Untreated and RANKL-treated samples were used as negative and positive differentiation controls, respectively, while RIG CM, added in the same range of volumes as RIG_LDH-GA CM, served as the experimental negative control. OCs are multinucleated cells characterized by a ring of intracellular actin filaments that seals the bone resorption lacuna. Thus, to visualize the differentiated phenotype induced by RANKL in RAW 264.7 macrophages and to quantitatively assess OC number, nuclei and actin filaments were stained with fluorescent Hoechst and TRITC-conjugated phalloidin, respectively. For each sample, the number of OCs was counted across the entire culture well. Compared to the positive control treated with RANKL alone, OC formation was inhibited in a dose-dependent manner by RIG_LDH-GA CM (Fig. 2**A**), with the number of OCs progressively decreasing until no OCs were observed at the highest dose of RIG_LDH-GA CM. This effect was absent in the RIG CM control samples, which were tested in the same dose range and did not affect OC differentiation. Notably, when comparing RIG_LDH-GA CM to RIG CM, the reduction in OC number was highly significant (*p* < 0.0001) at all doses except the lowest one. Compared to the RANKL-differentiated control, the decrease in OC number induced by RIG_LDH-GA CM was highly significant (*p* < 0.0001) at all tested doses. The dose dependency of the RIG_LDH-GA effect is further illustrated by the concentration–response curve, which plots the variation in osteoclast count (expressed as a percentage relative to the RANKL control, considered 100 % of countable OCs) as a function of the micromolar GA concentration (Fig. 2**B**). The calculated IC₅₀ for GA released by RIG_LDH-GA CM was 10.610 ± 1.993 µM (Log IC_50_ = 1.026 ± 0.082 µM), with R² = 0.96. Importantly, multinucleated cells with actin rings were observed in the samples treated with RANKL alone (RL) and in combination with the RIG CM control (RL/RIG), but were completely absent in those cultured with the highest tested dose (50 µL/well) of RIG_LDH-GA CM (RL/RIG_LDH-GA) and in the untreated control (untr.) (Fig. 2**A** and 2**C**).

### 3.3 Inhibition of OC functions

One of the major markers of mature OCs is TRAP, a bone matrix-degrading enzyme whose activity can be detected by a brown-to-purple colorimetric reaction, both in the culture supernatant and at the intracellular level. Soluble TRAP activity was measured in the culture supernatant, and the potential existence of a dose-dependent effect of RIG_LDH-GA CM was evaluated.

**Fig. 3:**
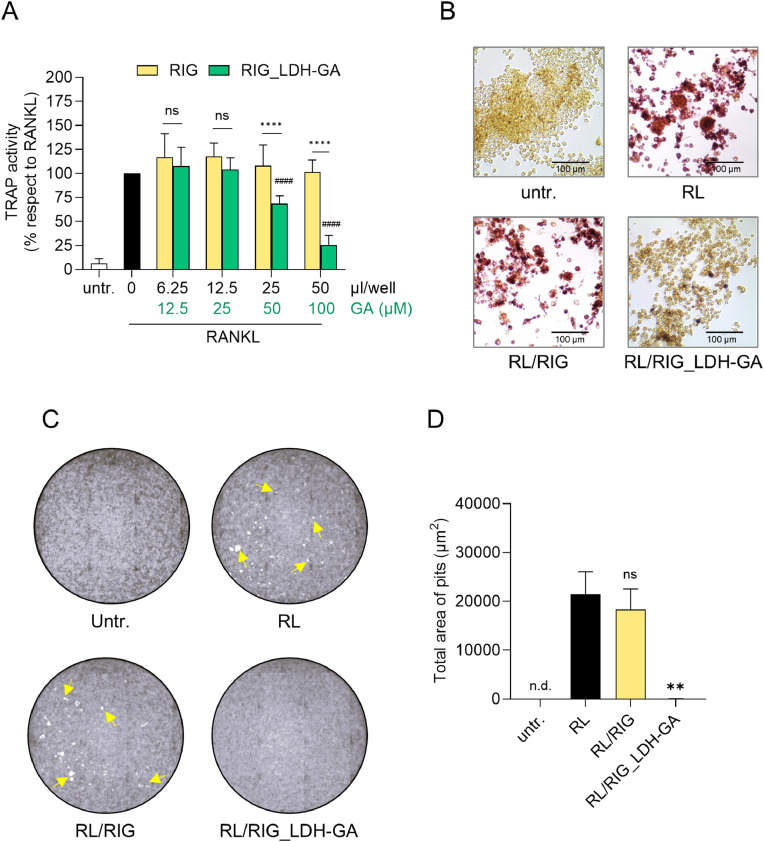
TRAP enzyme colorimetric reaction and bone resorption activity in cultures of RAW 264.7 cells treated with RANKL in combination with either RIG_LDH-GA (RL/RIG_LDH-GA) or RIG (RL/RIG) CM. (**A**) Quantitative analysis of TRAP activity in the culture supernatants of RAW 264.7 cells treated with RANKL (100 ng/mL) and a range of CM volumes. Data are represented as a percent of RANKL control (black bar) and reported as the mean ± SD of three independent experiments. For each sample treated with different volumes of RIG_LDH-GA CM, GA content is reported as the final concentration reached in the 96-well plate cell culture (100 μL/well). ns, not significant. Untr., untreated undifferentiated cells (white bar); ****, *p* < 0.0001, RANKL/RIG_LDH-GA-treated versus RANKL/RIG-treated (two-way ANOVA test); ^####^, *p* < 0.0001, RANKL/RIG_LDH-GA-treated versus RANKL-differentiated control (one-way ANOVA test). (**B**) Staining of TRAP-positive OCs induced from RAW 264.7 macrophages by RANKL alone (RL) or in combination with the maximum dose (50 µL/well, within a total volume of 100 µL per well) of either RIG_LDH-GA (RL/RIG_LDH-GA) or RIG (RL/RIG) CM; untr., undifferentiated and untreated undifferentiated cells. OCs are TRAP-positive purple-pink multinucleated cells having three or more nuclei. Representative images from one of three independent experiments. Images were captured under a bright-field light microscope EVOS® FL Auto Imaging System. (**C**) RAW 264.7 cells, seeded on CaP pre-coated wells of a 96-well plate (200 μL/well), were differentiated for 6 days in the presence of RANKL (100 ng/mL) alone (RL) or in combination with the maximum dose (100 µL/well, within a total volume of 200 µL per well) of either RIG or RIG_LDH-GA CM (RL/RIG or RL/RIG_LDH-GA, respectively). Untr., undifferentiated and untreated cells. Representative bright-field full-well images are reported. Lacunae are white areas; some of them are indicated by arrows. (**D**) Bar graph of quantitative analysis of total area of pits. Data are represented as the mean ± SD of two replicates. **, *p* = 0.0054, RIG or RIG_LDH-GA-treated versus RANKL-differentiated control (one-way ANOVA test).

As expected, at every tested dose, RIG CM did not affect the TRAP activity observed in the RANKL control, taken as reference with 100 % TRAP activity. In contrast, RIG_LDH-GA CM significantly reduced TRAP activity levels compared to both the RANKL positive control (25 µL and 50 μL, corresponding to a final GA concentration of 50 and 100 μM, *p* < 0.0001) and the RIG CM experimental control (25 µL and 50 µL, *p* < 0.0001), showing a dose-dependent trend starting from 12.5 µL (Fig. 3**A).** Intracellular TRAP activity was assessed by staining RAW 264.7 cells incubated for 5 days with RANKL and 50 µL of either RIG_LDH-GA (RL/RIG_LDH-GA) or RIG (RL/RIG) CM. Untreated (untr.) and RANKL-treated (RL) cells were used as negative and positive controls, respectively (Fig. 3**B**). The purple staining not only confirmed the results of TRAP activity measured in the culture supernatants but also allowed visualization of whether TRAP-positive cells were OCs—that is, multinucleated cells with three or more nuclei. The staining revealed the presence of mature OCs in both controls, RL and RL/RIG, but not in the untreated or RL/RIG_LDH-GA samples. Although RL/RIG_LDH-GA showed some TRAP-positive cells, only a few were multinucleated, indicating that—unlike the RL/RIG control—RIG_LDH-GA CM is effective in inhibiting the cell fusion process triggered by RANKL for the generation of mature OCs (Fig. 3**B**).

The main function of OCs is bone resorption activity. This can be evaluated in vitro by seeding cells in CaP pre-coated wells. If the culture conditions allow RAW 264.7 cells to differentiate into OCs, the coating matrix is locally eroded within the resorption lacunae of newly formed OCs, and pits can be visualized by brightfield microscopy. Consistent with the results on F-actin ring formation, RIG_LDH-GA CM, used at a 1:2 dilution of the total culture volume, completely suppressed the RANKL-induced osteoclast-mediated bone resorption observed in the RANKL/RIG CM co-cultured control (Fig. 3**C**). In fact, while pits were wide and frequent in wells treated with RANKL alone (RL) or in combination with RIG CM (RL/RIG), the coating surface remained intact in wells treated with RANKL in combination with RIG_LDH-GA CM (RL/RIG_LDH-GA), resembling the untreated RAW 264.7 control (untr.). Quantification of eroded areas using ImageJ software revealed a significant reduction in coating matrix resorption when comparing the RANKL/RIG_LDH-GA sample to the RANKL/RIG control (*p* = 0.0054) (Fig. 3**D**).

Thus, results from OC count, TRAP staining, and bone resorption activity collectively demonstrate that RIGENERA BTK BCP material functionalized with LDH-GA can effectively release the bioactive compound GA, which is capable of inhibiting OC formation.

### 3.4 Expression analysis of OC marker genes

**Fig. 4:**
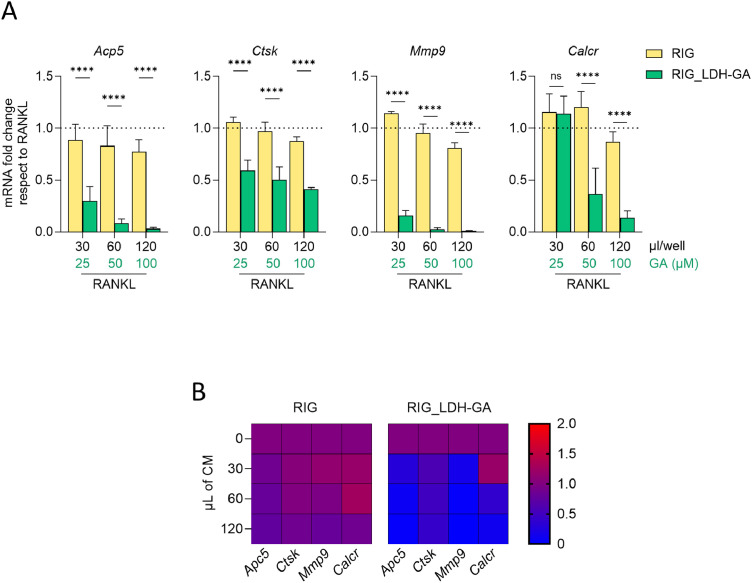
Real-time PCR analysis of OC marker transcripts in cultures of RAW 264.7 cells treated with RANKL in combination with either RIG (RL/RIG) or RIG_LDH-GA (RL/RIG_LDH-GA) CM. For each sample treated with different volumes of RIG_LDH-GA CM, GA content is reported as the final concentration reached in the 48-well plate cell culture (240 μL/well). (**A**) *Acp5, Ctsk, Mmp9*, and *Calcr* transcripts were normalized to the expression of *Gapdh* and reported as fold change of RIG- or RIG_LDH-GA-treated samples with respect to the normalized RANKL-differentiated calibrator (dotted line, fold change = 1). ****, *p* < 0.0001; RANKL/RIG- vs. RANKL/RIG_LDH-GA-treated (two-way ANOVA test). Data are represented as the mean ± SD of two replicates. (**B**) In the same settings of (**A**), the gene expression data are represented as a heat map. For each indicated volume of RIG or RIG_LDH-GA CM, the colour scale indicates the level of fold change of RANKL/RIG- or RANKL/RIG_LDH-GA-treated samples with respect to the positive control treated with RANKL alone (blue < 1, red ≥ 1).

The differentiation process induced by RANKL in macrophages includes the upregulation of osteoclastogenesis marker genes. Thus, to confirm at the molecular level the inhibitory effect of RIG_LDH-GA CM on OC formation, a comparative gene expression analysis was performed by real-time PCR in comparison to RIG CM. RANKL-treated RAW 264.7 cells were stimulated for 5 days in a 48-well plate (240 µL/well) with 30, 60, or 120 µL of either RIG or RIG_LDH-GA CM, the latter giving a final GA concentration of 25, 50 or 100 μM (Fig. 4**A**). For all tested doses, expression of the TRAP-encoding gene *Acp5* was significantly reduced compared to the RIG CM control (30, 60, and 120 µL, *p* < 0.0001), showing a dose-dependent decrease down to very low expression levels at 120 µL of RIG_LDH-GA CM. A similar trend was observed for *Mmp9*, the gene encoding matrix metalloproteinase-9 (30, 60, and 120 µL, *p* < 0.0001). The *Ctsk* gene, which encodes a lysosomal cysteine protease, was also significantly downregulated in a dose-dependent manner, although to a lesser extent (30, 60, and 120 µL, *p* < 0.0001). In contrast, significant repression of the calcitonin receptor gene *Calcr* was observed only at the two highest doses (60 and 120 µL, *p* < 0.0001).The heat map representation of gene expression analysis highlights the dose-dependent modulation of osteoclastogenesis marker genes (Fig. 4**B**) and underscores the efficacy of RIG_LDH-GA CM in inhibiting the expression of key genes involved in OC function. Western blot analysis confirmed the transcript regulation data (Fig. S3). Although the most pronounced decrease was observed for TRAP, the results for Ctsk, Mmp9, and Calcr proteins were also consistent with the overall downregulation pattern detected in the gene expression analysis.

### 3.5 RANK expression and signal transduction

**Fig. 5:**
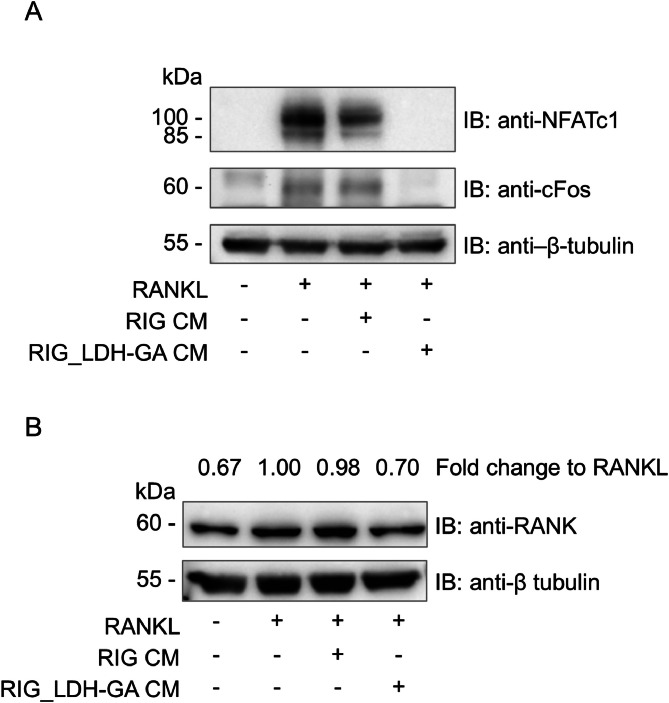
Modulation of RANK signaling pathway. (**A**) NFATc1 and c-Fos protein expression was assessed in RAW 264.7 cells cultured in 48-well plate (240 μL/well), either untreated or after 5-day treatment with RANKL (100 ng/mL), alone or in combination with either RIG or RIG_LDH-GA CM (120 μL/well). β-tubulin was analyzed as a loading control. (**B**) For RANK immunoblotting analysis, RAW 264.7 cells, cultured in 48-well plate (500 μL/well), were treated with RANKL (100 ng/mL) in the absence or in the presence of either RIG_LDH-GA or RIG CM (250 μL/well) for 24 h. RANK expression was normalized to β-tubulin, used as a loading control. Fold changes of untreated, RANKL/RIG- or RANKL/RIG_LDH-GA-treated vs. the positive control treated with RANKL alone are reported. One representative experiment of three.

The differentiation process from macrophages toward OCs is triggered by the binding of RANKL to its receptor RANK. Besides other mediators, the cascade of activated signals also includes the transcription factors c-Fos and NFATc1. This transduction pathway leads to *RANK* upregulation in a positive amplification loop, rendering the cell more and more sensitive to the RANKL differentiation signal [43]. To evaluate the possible inhibitory effect of RIG_LDH-GA CM on the RANK protein expression, whole cell lysates were prepared from RANKL-stimulated RAW 264.7 cells cotreated with either RIG_LDH-GA CM or RIG CM as a control. Because of their late upregulation during the differentiation process transforming RAW 264.7 macrophages into OCs [43], c-Fos and NFATc1 were investigated after 5 days of culture with RANKL. As expected, both transcription factors were upregulated by RANKL treatment, either alone or in combination with RIG CM used as a control. In contrast, in the sample treated with RIG_LDH-GA CM, c-Fos and NFATc1 signals were almost absent and similar to those observed in the untreated sample (Fig. 5**A**). Twenty-four hours after the treatment with RANKL, the protein expression of the RANK receptor was investigated as well (Fig. 5**B**). In the sample treated with RANKL/RIG_LDH-GA CM, the immunoblot revealed a RANK signal as in untreated RAW 264.7 cells that is weaker than in RANKL-differentiated and RANKL/RIG CM-treated controls.

Thus, the downmodulation of c-Fos and NFATc1 transcription factors, persisting up to 5 days of culture, confirmed the interference of RIG_LDH-GA CM in the signaling pathway of the RANK receptor (a condition consistent with the observed decrease of RANK expression in RAW 264.7 cells already after 24 h of treatment with RIG_LDH-GA CM), rendering cells not sensitive to RANKL differentiation input by 5 days of treatment.

## 4 Discussion

Preserving alveolar bone volume after tooth loss is essential to ensure successful implant placement, particularly in cases where physiological bone remodeling leads to significant resorption. The choice of bone grafting material is therefore critical, as it influences not only osteoconductivity and regenerative potential but also the rate of resorption [44]. In this study, we aimed to enhance the biological performance of a BCP scaffold (RIGENERA BTK BCP), composed of HA and β-TCP, by functionalizing it with a ZnAl-LDH intercalated with GA. This approach was designed to combine the scaffold’s structural and osteoconductive properties with the antiresorptive and bioactive capabilities of a controlled-release system.

The choice of GA as a suitable molecule to be intercalated between the LDH lamellae was driven by two criteria: its anionic state at the cell culture pH of 7.4 [45] and its previously demonstrated antiosteoclastogenic activity [34,35]. In aqueous solution, GA, having four acidic protons, is present as a range of deprotonated species depending on the pH of the solution. When the pH is low (pH ≤ 4), the GA molecule with four protons is the dominant species. As the pH increases, the relative proportions of the protonated species change until almost all protons are donated at pH = 14. At pH = 7.4, the pH value of in vitro cell cultures, the most abundant GA species is the monoanionic one, that is GA without the carboxylic proton and with the three protons of the phenolic hydroxy groups. Thus, to prepare LDH intercalated with GA, deprotonation of GA’s carboxyl group was achieved by adding an equimolar amount of NaOH. Under these conditions, the monoanionic sodium gallate can exchange the acetate previously intercalated in the pristine LDH (Fig. 1**B**). The resulting coating material was tested for its ability to release GA upon incubation in a physiological solution; the chloride anions present were capable of exchanging GA, which was quantified in the incubation solution in a time-course experiment (Fig. 1**E**), indicating the feasibility of a drug delivery system made from RIGENERA BTK BCP scaffolds coated with LDH intercalated with GA.

LDH has already been assessed in several models optimizing the antioxidant and antibacterial activities of GA. As an inorganic host matrix for GA delayed release, MgAl-LDH solid was found to be effective in protecting the antioxidant drug from oxidation during storage prior to application. When released from LDH in a controlled manner, GA served as an excellent antioxidant to scavenge free radicals [46]. Another study evaluated the anti-biofilm effectiveness of a novel nanocomposite of ZnAl-LDH intercalated with GA as a chelating agent and demonstrated its efficiency as an antimicrobial agent and/or sanitizer inhibiting the growth of potentially pathogenic bacteria causing bovine clinical mastitis [47]. Moreover, carboxymethyl cellulose films loaded with GA-intercalated ZnAl-LDH, developed for wound-healing applications, exhibited the desired antibacterial activity against both Gram-positive and Gram-negative microorganisms, with the highest bactericidal effect on *Staphylococcus aureus* [48].

In our study, LDH-GA was used as a coating agent for RIGENERA BTK BCP blocks to create a drug delivery system integrated into a bone substitute material. The incubation of RIG_LDH-GA in culture medium mimicked the fluidic environment of a dental socket, where the composite could be placed to counteract alveolar bone loss and enhance the osteogenic process. The CM obtained by this procedure was tested in an in vitro cell model of RANKL-induced differentiation to assess whether the GA released would modulate the differentiation process of RAW 264.7 macrophages into OCs, the cells critically involved in bone erosion. The observed effect was dose-dependent and strikingly inhibited the key events leading to the fusion of pre-osteoclasts into giant multinucleated bone-resorbing cells. The most indicative result of the inhibitory effect exerted by RIG_LDH-GA CM on RAW 264.7 differentiation into OCs was the decreased number of fused cells observed at lower doses, with complete inhibition detected at the highest tested dose (Fig. 2**A**). OCs counting was performed by fluorescent staining of characteristic morphological markers of OCs, which include nuclei (by definition, more than three) derived from the fusion process of mononucleated precursors and the intracellular actin ring sealing the bone resorption lacuna [6,49]. OCs differentiated from macrophages typically express the TRAP enzyme, a marker of bone resorption activity that can be detected through a colorimetric reaction both in the culture supernatant and at the intracellular level. However, the expression of the TRAP enzyme is a necessary but not sufficient condition to define a cell as an OC, as the fusion of cells to produce multinucleated structures must also occur. In fact, weak or inhibited RANKL stimulation (as observed upon treatment with RIG_LDH-GA CM) may sustain poor TRAP expression in responding cells but not support the fusion process (Fig. 3**B**). Indeed, the TRAP-encoding *Acp5* gene and other OC marker genes tested were downregulated in a dose-dependent manner by RIG_LDH-GA CM, clearly suggesting that the GA-releasing system is effective in targeting molecular pathways that regulate the expression of proteases involved in extracellular matrix and bone resorption *(Mmp9, Acp5,* and *Ctsk*) and the regulation of OC-mediated bone resorption (*Calcr* gene) (Fig. 4**A**).

During the OC differentiation process, a feedforward amplification loop is activated, involving the RANK receptor and the transcription factor NFATc1. Ligation of RANK by RANKL activates several signaling pathways, including MAPKs, AKT, and NF-κB, which lead to the induction of OC-specific genes such as *Nfatc1, Ctsk*, and *Acp5* [50]. In particular, the RANKL-dependent downstream nuclear translocation of c-Fos induces *Nfatc1* and thus triggers a sustained NFATc1-dependent transcriptional program during OC differentiation [43]. Among other key genes involved in OC function, NFATc1 induces the RANK gene by directly binding to its specific binding sequence in the RANK promoter [51]. The positive gene expression feedback loop between *NFATc1* and *RANK* accelerates the terminal differentiation of RANK-positive committed precursors into mature osteoclasts [43]. In our study, the negative regulation of *c-Fos* expression in RAW 264.7 cells by RIG_LDH-GA CM significantly disrupts the expression of *NFATc1*, the master regulator of OC differentiation, and consequently *RANK* as well (Fig. 5**A** and 5**B**). Therefore, the absence of the RANKL-dependent amplification loop between NFATc1 and RANK prevents RAW 264.7 cells from acquiring a mature OC phenotype.

In our study the ZnAl-LDH coating was primarily designed as a carrier for sustained GA release, However, we also considered the potential independent effects that Zn²⁺ or Al³⁺ ions released from the matrix could exert on OC-mediated bone resorption. ICP-OES analysis performed under the same conditions used for GA release profiling showed that both Zn²⁺ and Al³⁺ concentrations remained below the instrument's detection limits (0.5–1 ppb, equivalent to ∼7.5–15 nM for Zn²⁺ and ∼18.5–37 nM for Al³⁺). These levels are several orders of magnitude lower than those reported to influence OC or OB activity, which typically occur at concentrations in the hundreds of μM [[52], [53], [54], [55]]. Therefore, it is unlikely that the released ions have any appreciable direct effect on bone cell function. Nonetheless, the LDH matrix may indirectly contribute by enhancing GA stability, site-specific retention, and bioavailability, potentially improving the overall efficacy of the system.

Compared to other bone grafting systems reported in the literature, the ZnAl-LDH_GA-functionalized RIGENERA BTK BCP offers a unique combination of structural support and targeted biological activity. While traditional BCP scaffolds provide excellent osteoconductivity, they lack active control over bone resorption and are not designed for drug delivery [56,57]. Other systems incorporating bioactive molecules such as alendronate, resveratrol, or curcumin have shown potential in modulating osteoclast activity but often suffer from burst release, limited stability, or unspecific systemic effects [[58], [59], [60]]. In contrast, our approach leverages the LDH matrix to achieve a sustained and localized release of GA, a naturally occurring antiosteoclastogenic molecule with antioxidant properties [34,35,61]. This controlled delivery results in a dose-dependent inhibition of key OC differentiation markers, suggesting a more effective and selective modulation of bone remodeling. Moreover, the absence of detectable levels of Zn²⁺ and Al³⁺ ions released under experimental conditions reduces concerns about potential cytotoxicity or unintended biological effects, as biologically relevant effects typically require concentrations in the micromolar range [53,55]. These features position our system as a promising multifunctional platform for alveolar bone preservation, although further in vivo studies will be necessary to validate its long-term efficacy and biocompatibility.

## 5 Conclusions

Alveolar bone loss is a significant concern in periodontitis and following dental extractions due to excessive OC bone-resorbing activity not properly counterbalanced by the OB-mediated bone formation process. In the present study, the synthetic bone substitute RIGENERA BTK BCP, composed of HA and β-TCP, was coated with ZnAl-based LDH intercalated with GA to obtain a drug delivery system capable of locally releasing the antiosteoclastogenic molecule GA when placed in the socket. The functionalized material effectively released GA from the LDH matrix and enabled the preparation of a CM to be tested in vitro using a RANKL-induced OC differentiation model. The GA-containing CM efficiently inhibited, in a dose-dependent manner, OC formation; TRAP enzyme expression; OC marker gene expression; bone resorption activity; and the expression of c-Fos, NFATc1, and the RANK receptor, all essential for the OC differentiation process. Thus, the functionalization of RIGENERA BTK BCP with LDH and GA represents an osteoprotective enhancement of this biomaterial, which already possesses bone regenerative properties, and could hold promising clinical potential in preventing OC-mediated alveolar bone loss.

## Abbreviations

ACE, acetate; BCP, biphasic calcium phosphate, β-TCP, β-tricalcium phosphate; CaP, carbonate apatite; CM, conditioned medium; FBS, fetal bovine serum; FT-IR, Fourier Transform Infrared Spectroscopy; GA, gallic acid; HA, hydroxyapatite; ICP-OES, Inductively Coupled Plasma–Optical Emission Spectrometer; LDH, layered double hydroxide; LDH-ACE, layered double hydroxide loaded with acetate; LDH-GA, layered double hydroxide loaded with gallic acid; LDH—NO_3,_ layered double hydroxide loaded with nitrate; MTT, 3-(4,5-dimethylthiazol-2-yl)-2,5-diphenyltetrazolium bromide; OBs, osteoblasts; OCs, osteoclasts; RANK, Receptor Activator of Nuclear factor Kappa-Β; RANKL, Receptor Activator of Nuclear factor Kappa-Β Ligand; RIG, unfunctionalized RIGENERA BTK BCP; RIG_LDH-GA, RIGENERA BTK BCP functionalized with layered double hydroxide and gallic acid; SEM, scanning electron microscope; TRAP, tartrate-resistant acid phosphatase; XRPD, X-ray powder diffraction.

## Funding

This work was funded by the European Union – NextGenerationEU under the Italian Ministry of University and Research (MUR) National Innovation Ecosystem grant ECS00000041 - VITALITY - CUP J97G22000170005.

## CRediT authorship contribution statement

**Chiara Suvieri:** Writing – review & editing, Visualization, Investigation, Formal analysis, Data curation. **Maria Bastianini:** Writing – original draft, Methodology. **Stefano Pagano:** Writing – review & editing, Resources. **Lorella Marinucci:** Methodology. **Valeria Ambrogi:** Writing – review & editing. **Leonardo Leonardi:** Software. **Carmela Conte:** Investigation. **Maria Teresa Pallotta:** Writing – review & editing, Validation. **Bernard Fioretti:** Investigation. **Giovanna Traina:** Software. **Michele Sisani:** Methodology. **Maria Laura Belladonna:** Writing – original draft, Visualization, Supervision, Project administration, Funding acquisition, Conceptualization.

## Declaration of competing interest

The authors declare that they have no known competing financial interests or personal relationships that could have appeared to influence the work reported in this paper.

## Data Availability

Data will be made available on request.

## Appendix Supplementary materials

XRPD of the RIGENERA BTK BCP block unfunctionalized (RIG) and functionalized with LDH_GA (RIG_LDH-GA). Image, image 1

MTT analysis of cell viability in cultures of RAW 264.7 cells seeded in 96-well plate (100 μL/well) and treated with RANKL and RIG_LDH-GA or RIG CM. Cell viability at the indicated volumes of the RIG_LDH-GA or RIG CM was evaluated by MTT assay; it is reported as a percentage of viable cells with respect to RANKL-differentiated control. Dotted lines indicate 100% and 75% control viability. For each sample treated with different volumes of RIG_LDH-GA CM, GA content is reported as the final concentration reached in the cell culture. Untr., undifferentiated and untreated cells (white bar); treatment with RANKL alone (100 ng/mL) is indicated by the black bar; cotreatment with RANKL and CMs is indicated by colored bars. Data are mean values ± SD of three independent experiments. For RANKL-treated cells, CM-treated (colored bars) versus untreated cells (black bars) were analyzed by a one-way ANOVA test. Image, image 2

Protein expression analysis of OC markers in RAW 264.7 cells cultured for 5 days in a 48-well plate (240 μL/well) and treated with RANKL (100 ng/mL) alone or in combination with either RIG or RIG_LDH-GA CM at maximum concentration (120 μL/well). Cells were recovered to prepare whole cell lysates as described in paragraph 2.12. Protein expression was normalized to β-tubulin, used as a loading control. For RANKL/RIG and RANKL/RIG_LDH-GA samples fold changes relative to the positive control treated with RANKL alone are reported. One representative experiment. The following primary antibodies were used: rabbit Acp5 polyclonal antibody targeting TRAP protein (#PA5-106914, Invitrogen, Carlsbad, CA, USA), rabbit Ctsk polyclonal antibody (#PA5-102483, Invitrogen, Carlsbad, CA, USA), mouse Mmp9 (5G3) monoclonal antibody (#MA5-15886, Invitrogen, Carlsbad, CA, USA) rabbit Calcr polyclonal antibody (#20868-1-AP, Proteintech, Rosemont, IL, USA) and mouse β-tubulin (AA2) monoclonal antibody (#T8328, Sigma Aldrich, St. Louis, MO, USA); n.d.: not detected. Image, image 3

## Footnotes

Supplementary material associated with this article can be found, in the online version, at doi:10.1016/j.bbiosy.2025.100119.
