# Supplementary figures and images for: Gallic acid released by a layered double hydroxide-coated scaffold of hydroxyapatite and β-tricalcium phosphate inhibits the osteoclast formation In Vitro
Source: Biomater Biosyst. 2025 Aug 20;19:100119. doi: 10.1016/j.bbiosy.2025.100119 (PMC12398821; doi:10.1016/j.bbiosy.2025.100119)

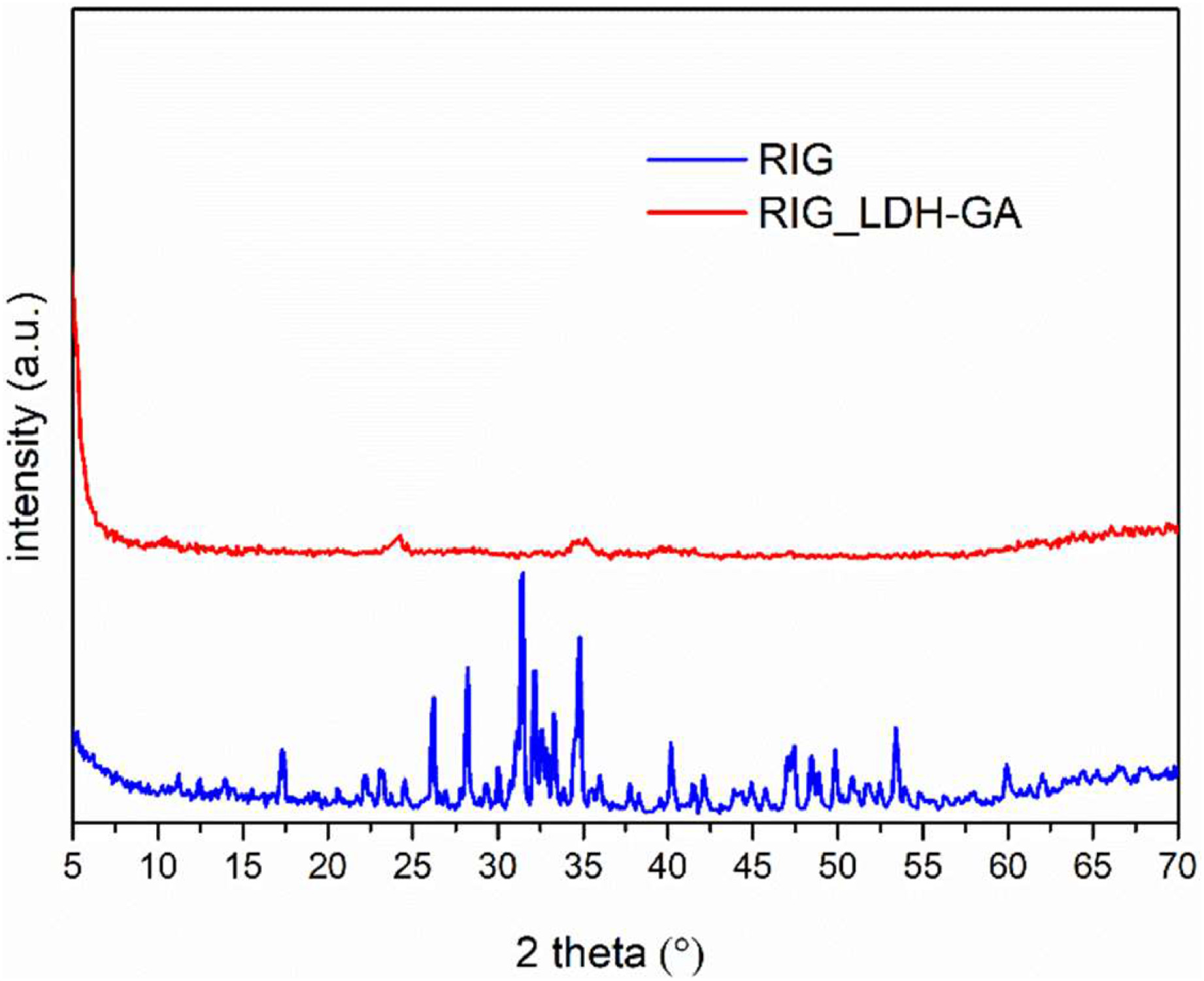

Supplement: Supplementary file 1 — XRPD of the RIGENERA BTK BCP block unfunctionalized (RIG) and functionalized with LDH_GA (RIG_LDH-GA). [file mmc1.jpg]

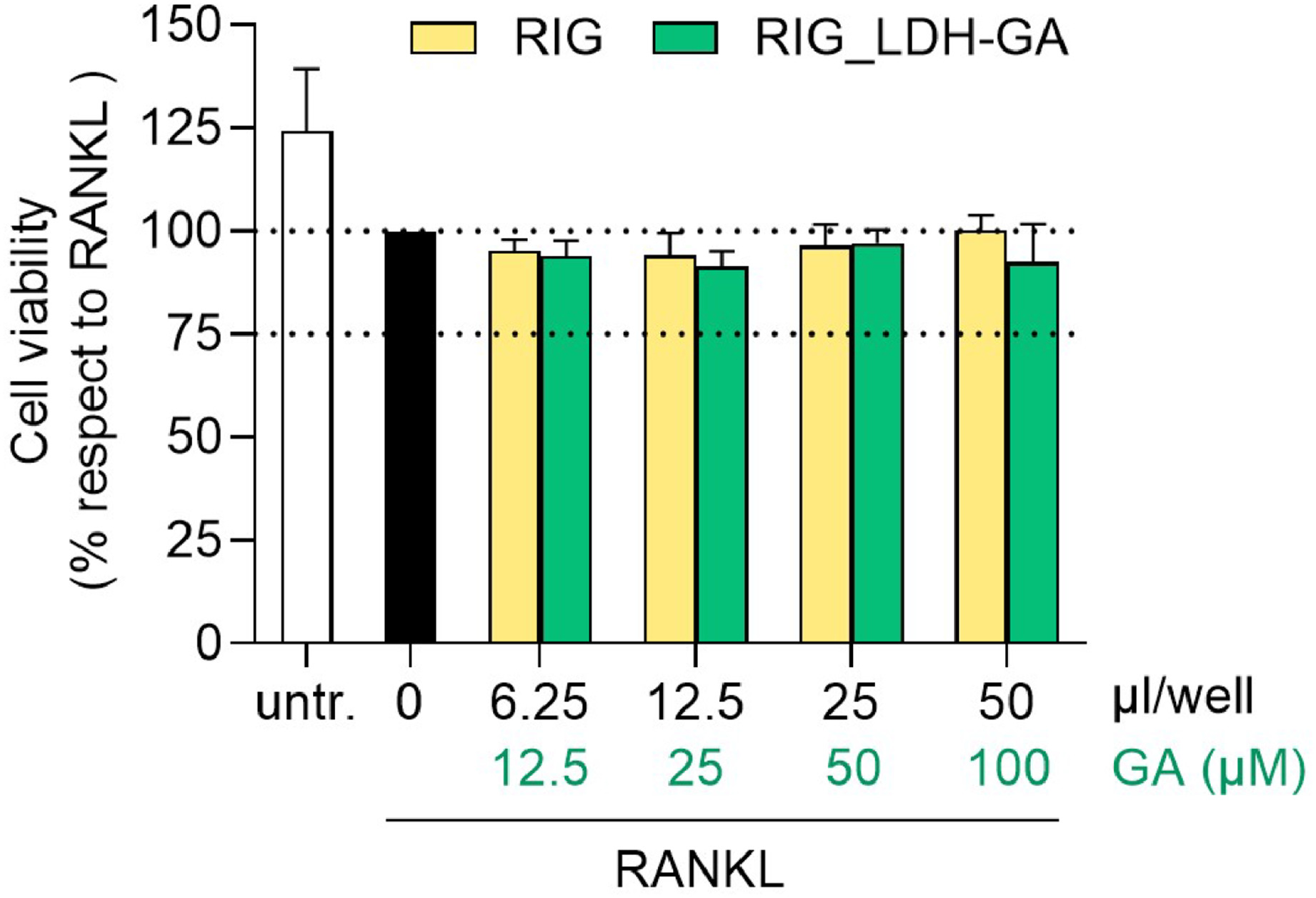

Supplement: Supplementary file 2 — MTT analysis of cell viability in cultures of RAW 264.7 cells seeded in 96-well plate (100 μL/well) and treated with RANKL and RIG_LDH-GA or RIG CM. Cell viability at the indicated volumes of the RIG_LDH-GA or RIG CM was evaluated by MTT assay; it is reported as a percentage of viable cells with respect to RANKL-differentiated control. Dotted lines indicate 100% and 75% control viability. For each sample treated with different volumes of RIG_LDH-GA CM, GA content is reported as the final concentration reached in the cell culture. Untr., undifferentiated and untreated cells (white bar); treatment with RANKL alone (100 ng/mL) is indicated by the black bar; cotreatment with RANKL and CMs is indicated by colored bars. Data are mean values ± SD of three independent experiments. For RANKL-treated cells, CM-treated (colored bars) versus untreated cells (black bars) were analyzed by a one-way ANOVA test. [file mmc2.jpg]

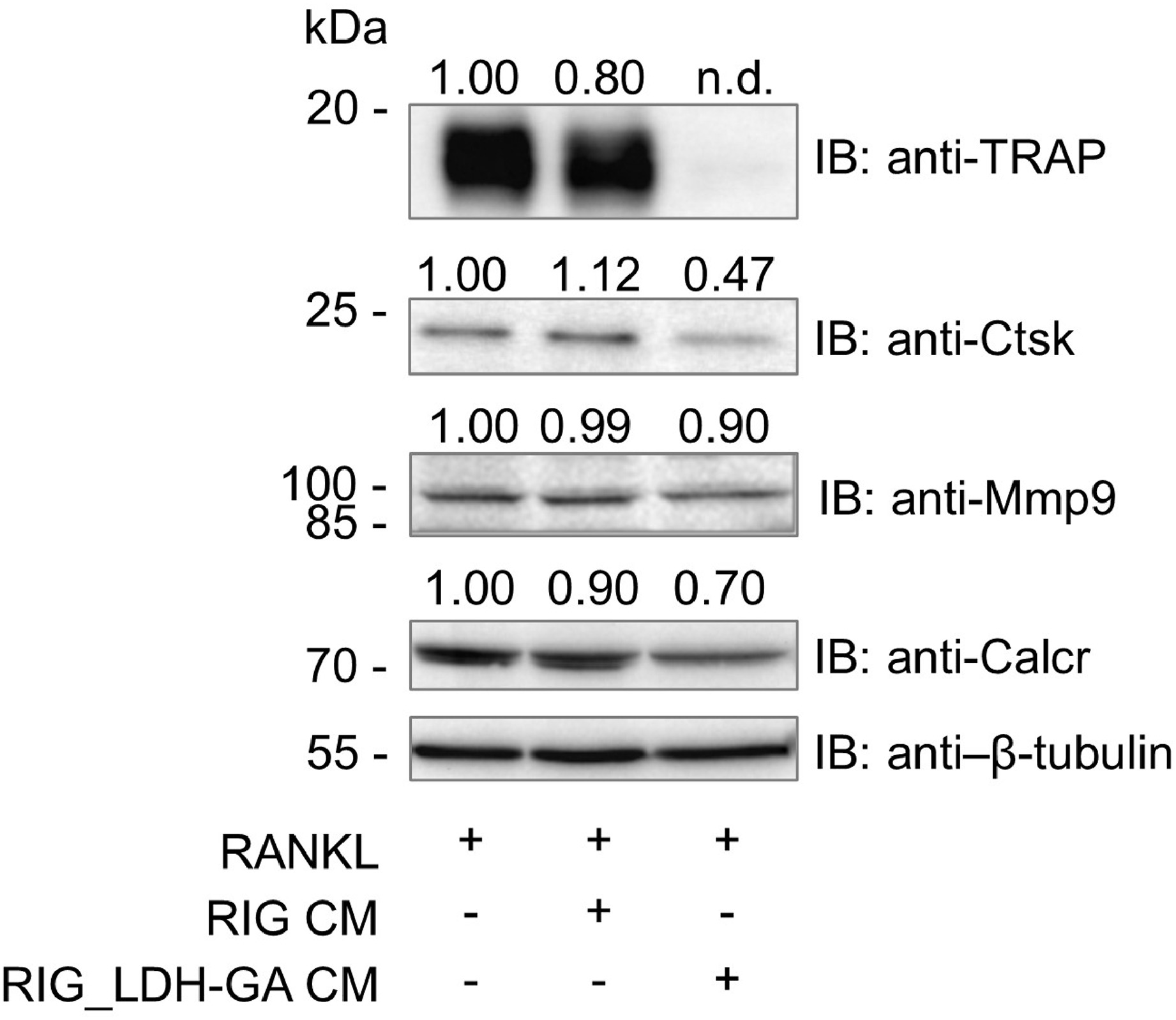

Supplement: Supplementary file 3 — Protein expression analysis of OC markers in RAW 264.7 cells cultured for 5 days in a 48-well plate (240 μL/well) and treated with RANKL (100 ng/mL) alone or in combination with either RIG or RIG_LDH-GA CM at maximum concentration (120 μL/well). Cells were recovered to prepare whole cell lysates as described in paragraph 2.12. Protein expression was normalized to β-tubulin, used as a loading control. For RANKL/RIG and RANKL/RIG_LDH-GA samples fold changes relative to the positive control treated with RANKL alone are reported. One representative experiment. The following primary antibodies were used: rabbit Acp5 polyclonal antibody targeting TRAP protein (#PA5-106914, Invitrogen, Carlsbad, CA, USA), rabbit Ctsk polyclonal antibody (#PA5-102483, Invitrogen, Carlsbad, CA, USA), mouse Mmp9 (5G3) monoclonal antibody (#MA5-15886, Invitrogen, Carlsbad, CA, USA) rabbit Calcr polyclonal antibody (#20868-1-AP, Proteintech, Rosemont, IL, USA) and mouse β-tubulin (AA2) monoclonal antibody (#T8328, Sigma Aldrich, St. Louis, MO, USA); n.d.: not detected. [file mmc3.jpg]
